# Supplementary material for: Complexome profiling of the Chlamydomonas psb28 mutant reveals TEF5 as an early PSII assembly factor
Source: Plant Cell. 2025 Mar 17;37(6):koaf055. doi: 10.1093/plcell/koaf055 (PMC12164590; doi:10.1093/plcell/koaf055)
Supplement: koaf055_Supplementary_Data [file koaf055_supplementary_data.zip › psb28_interactive.zip!/plots/intensity/qualityPlots/peptides/_Cre03.g199150.t1.2.html]

### Cre03.g199150.t1.2

Trivial name:   
SeqCoverage [median]: 32.40  
Mass: 29.73 kDa  
  
 

| Sequence | MissedCleavage | Mass [Da] | wtIntensity | muIntensity | Score |
| --- | --- | --- | --- | --- | --- |
| AAAEEALR | 0 | 829.43 | 2971.30 | 10970.29 | 162.50 |
| AGTPPLASGR | 0 | 925.50 | 919.55 | 7238.59 | 109.44 |
| AHYPGTGVALRPGVIYGDR | 1 | 1998.04 | 931.27 | 7909.75 | 96.67 |
| GNALEPATYTEHLR | 0 | 1570.77 | 1994.45 | 8083.78 | 125.61 |
| LGPETAAR | 0 | 813.43 | 1420.85 | 0.00 | 101.28 |
| MAAVPLVGAAFVPPVSVETVAR | 0 | 2180.20 | 497.12 | 0.00 | 87.49 |
| TNGAANVALIEAAK | 0 | 1341.73 | 0.00 | 738.42 | 55.26 |
|  |  |  | 8734.5 | 34940.8 |  |
